# Inhibition of Notch1 Signaling at the Subacute Stage of Stroke Promotes Endogenous Neurogenesis and Motor Recovery After Stroke

**DOI:** 10.3389/fncel.2018.00245

**Published:** 2018-08-07

**Authors:** Xiao-Zhu Hao, Le-Kang Yin, Jia-Qi Tian, Chan-Chan Li, Xiao-Yuan Feng, Zhen-Wei Yao, Min Jiang, Yan-Mei Yang

**Affiliations:** ^1^Department of Radiology, Huashan Hospital, Fudan University, Shanghai, China; ^2^Department of Radiology, Shanghai Chest Hospital, Shanghai Jiaotong University, Shanghai, China; ^3^Institutes of Brain Science and State Key Laboratory of Medical Neurobiology, Fudan University, Shanghai, China

**Keywords:** Notch1, neurogenesis, MCAO, DAPT, striatum, internal capsule, magnetic resonance imaging, stroke

## Abstract

**Background and Purpose:** It is still not clear whether Notch1 signaling inhibition can promote functional outcomes after stroke, given that it plays time-dependent roles in the sequential process of endogenous neurogenesis. The purpose of this study was to identify the appropriate time frame for Notch1 signaling inhibition according to the temporal evolution of Notch1 signaling activation and the responses of neural stem cells (NSCs), in order to target it for therapeutic intervention and stimulate neurorestorative strategies after stroke.

**Methods:** Sprague-Dawley (SD) rats were subjected to 90-min of middle cerebral artery occlusion (MCAO). Rats were sacrificed before, and at day 1, day 2, day 3, day 4, and day 7 after ischemia for immunohistochemical analysis of the Notch intracellular domain (NICD), Nestin and doublecortin (Dcx). Next, MCAO rats were treated with the γ-secretase inhibitor *N*-[*N*-(3,5-di uorophenacetyl)-1-alanyl]-S-phenylglycine t-butylester (DAPT) or with saline at day 4 after ischemia, and subsequently evaluated with behavioral test analysis and magnetic resonance imaging (MRI). The rat brains were then harvested for immunohistochemical analysis of Dcx, NeuN and myelin basic protein (MBP) at 2, 3, 4, and 8 weeks.

**Results:** Notch1 signaling was maximally activated at day 3 after ischemia in parallel with the temporal evolution of NSCs. Inhibiting Notch1 signaling at day 4 after reperfusion with DAPT further promoted recovery of MRI parameters of the corticospinal tract (CST) and the functional outcomes, concomitantly with an increase in neuroblasts, their migration to the ischemic boundary, and potential differentiation to mature neurons, as well as the amelioration of axonal bundle integrity.

**Conclusion:** Inhibition of Notch1 signaling at the subacute stage of stroke could maximally promote endogenous neurogenesis and axonal reorganization.

## Introduction

Stroke is one of the leading causes of death and serious long-term disability (Stinear et al., 2007; Smajlović, 2015). Importantly, a large number of stroke patients are permanently disabled in that only a minority of patients can benefit from thrombolysis given its limited therapeutic time window. Novel neurorestorative therapies with a wider therapeutic window that can promote brain repair, are thus, urgently needed to enhance functional neurological recovery following stroke (Barone, 2010; Zhan et al., 2011). Previous studies have demonstrated that cerebral ischemia induces proliferation of NSCs in the SVZ, which migrate into the damaged brain regions, differentiate into mature neurons and ultimately integrate into local as well as remote neural circuits (Arvidsson et al., 2002; Zhang et al., 2008; Wang L. et al., 2009; Sun et al., 2013), suggesting that endogenous neurogenesis could be a target for rehabilitative therapy in stroke patients.

In recent years, Notch1 signaling, which is critical for endogenous neurogenesis, has been regarded as a potential therapeutic target for promoting functional recovery after stroke (Wei et al., 2011). Notch1 is expressed in NSCs and neuroblasts and its activity is fundamental for neural development as well as neural specification by controlling maintenance, proliferation and differentiation of NSCs in young and aged brain in normal or pathological conditions (Zhang et al., 2008; Wang X. et al., 2009; Sun et al., 2013). Interestingly, while some studies have found that Notch1 signaling activation could promote neurogenesis (Oya et al., 2009; Wang X. et al., 2009), others support the idea that Notch1 signaling negatively regulates neurogenesis (Li et al., 2012). Notably, preventing Notch1 cleavage into the Notch intracellular domain (NICD) with the γ-secretase inhibitor N-[N-(3,5-diuorophenacetyl)-1-alanyl]-S-phenylglycine t-butylester (DAPT), subsequently improves functional outcomes following stroke (Li et al., 2012). Except for differences in animal strain and stroke models, the most plausible explanation for the conflicting results cited above is the spatio-temporal regulation of Notch activity (Zhao et al., 2012); in other words, Notch-1 signaling playing space and time-dependent roles in the sequential process of neurogenesis (Chambers et al., 2001). Moreover, several studies have found that Notch-1 signaling was activated in the acute stage of stroke to promote NSCs proliferation and was attenuated in the subacute stage to promote neuronal differentiation (Oya et al., 2009; Wang L. et al., 2009). Based on this standpoint, the detection of the temporal evolution of Notch1 signaling activation following cerebral ischemia and attempts to timely control its activation are required to augment the neural progenitor pool and promote neural differentiation to attain morphological and functional maturity in the adult brain.

Furthermore, it appears unreasonable and insufficient to define the beneficial or detrimental effects of therapeutic interventions of the Notch1 pathway based on *in vitro* pathological examinations. Thus, it is important to develop non-invasive methods to monitor modifications of brain tissue and predict long-term motor outcomes, which is essential to promote clinical applications of emerging neurorestorative therapies. Cross-sectional studies have demonstrated that diffusion tensor imaging (DTI) could provide unparalleled insights into the microstructural properties of central nervous system (CNS) tissue (Nucifora et al., 2007; Budde and Frank, 2012). For instance, diffusion parameter changes in the CST have been established as surrogate makers of motor deficit after stroke (Thomalla et al., 2005; Stinear et al., 2007; Lindenberg et al., 2012; Feng et al., 2015).

In this study, firstly, we aimed to detect the temporal evolution of Notch1 signaling activation and NSCs responses after stroke. Based on our results, we then attempted to find the appropriate therapeutic time frame for DAPT treatment. More importantly, for the first time, we measured the comprehensive microstructure changes in the CNS with a set of MR parameters, combined with the post mortem immunohistochemical analysis of neurogenesis and remyelination of the CST, and ultimately demonstrated the neurorestorative effects of DAPT treatment at the subacute stage after stroke.

## Materials and Methods

### Animals

Adult male Sprague-Dawley rats (260–270 g) were obtained from the Shanghai Experimental Animal Center of Fudan University. All procedures performed in this study were approved by the Fudan University of Institutional Animal Care and Use Committee, and every effort was made to minimize suffering and to reduce the number of animals used (20171740A704).

### Experimental Groups

For semiquantitative evaluation of Notch1 signaling activation and neural proliferative response at the early stage of stroke, rats were subjected to MCAO. Thirty-eight rats underwent the surgery and eight rats were excluded from our analysis because of death or poor lesions which is shown in the T2-weighted MR images 24 h after surgery (data not shown). In our study, five rats were sacrificed respectively before, and at day 1, day 2, day 3, day 4, and day 7 after the induced stroke, and subjected to immunohistochemical analysis of Nestin, Dcx, and the NICD.

**FIGURE 1:**
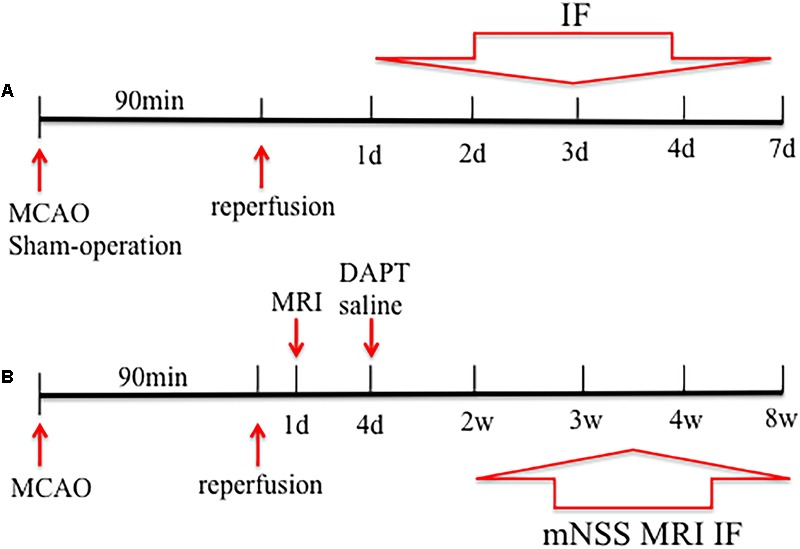
Study design of the experiment. Rats were subjected to middle cerebral artery occlusion (MCAO) in **(A,B)**. **(A)** Five rats were sacrificed before and 1, 2, 3, 4, and 7 days after stroke, and subjected to immunohistochemical analysis. **(B)** Rats were treated by DAPT or saline at day4, and eight rats of each subgroup were successively performed with behavioral test, magnetic resonance imaging (MRI) and the subsequent decapitation for immunohistochemical analysis 2, 3, 4, and 8 weeks after stroke.

To evaluate proliferative responses following treatment with the γ-secretase inhibitor, DAPT, at the subacute stage of stroke, rats were subjected to MCAO, treated by intracerebroventricular (i.c.v) administration (day 4) of DAPT or saline. Forty rats underwent surgery, of which three rats were not selected for further experiments because of poor lesions and five rats died during the course of our study. Therefore, eight rats of each subgroup were successively evaluated with behavioral tests and MRI, followed by tissue harvesting for immunohistochemical analysis of Dcx, NeuN, and MBP at 2, 3, 4, and 8 weeks respectively. **Figure 1** shows the time schedule for experimental procedures in all groups (**Figure 1**).

### Stroke Model

Rats were anesthetized with an intraperitoneal (i.p.) injection of 10% chloral hydrate under spontaneous inspiration with continuous body temperature monitoring 37°C ± 0.5°C, during the surgical procedures. For all rats, the left middle cerebral artery (MCA) was occluded for 90 min. In details, rats were immobilized by a tooth holder and with binding of all limbs, followed by the insertion of a 4.0 silicon-coated polypropylene suture into the left internal carotid artery (ICA) through the external carotid artery (ECA) and CCA to block blood flow to the MCA. After 90 min, the filament was withdrawn from the ICA to allow reperfusion. The follow-up T2 MRI was acquired under the anesthetized circumstances to check the occlusion of the left hemisphere 24 h post-stroke. The above procedure was identical to that of our previous study (Hao et al., 2016).

### Administration of γ-secretase Inhibitor

DAPT powder (Sigma-Aldrich, St. Louis, MO, United States), was dissolved in DMSO to prepare concentrations of 8.3 mg/ml. DAPT solution (0.03 mg/kg) was stereotactically injected into the lateral cerebral ventricle (LV) for the DAPT treated group rats 4 days after the induced stroke. Rats were anesthetized and placed in a stereotactic holder and immobilized by earplugs and a tooth holder. A burr hole was drilled in the skull, 4.0 mm deep into the pial surface, -2.0 mm anteroposterior relative to the bregma, and 1.0 mm lateral to the midline, according to Paxinos and Watson (1998). With a 2.0 μl Hamilton syringe, DAPT administration was finished within 3 min and the needle was left in place for 4 min to prevent leakage along the injection track. Animals in the control group received the same volume of saline at the same time point.

### Magnetic Resonance Imaging

Multimodal MRI of subject animals, including relaxation time imaging and diffusion imaging, was temporally conducted in each experimental group. Prior to MRI, animals were anesthetized by the same procedure as described for the MCAO model (see above). The body temperature was continuously monitored at 37°C ± 0.5°C, at the same time, blood oxygen saturation and heart rate were also monitored during MRI procedures. The MRI measurements were performed in a 3.0-T horizontal magnet (Discovery MR750, GE Medical Systems, Milwaukee, WI, United States) with a 60-mm-diameter gradient coil (Magtron Inc., Jiangyin, China).

T2-weighted MR images were acquired by a fast spin-echo sequence with the following parameters: TR/TE = 4,000 ms/96 ms, scan time = 3 min, FOV = 6 cm × 6 cm, matrix = 256 × 256, ST = 1.8 mm, spatial resolution = 0.24 mm × 0.24 mm × 1.8 mm, inter-slice distance = 2 mm, number of slices = 15, and NA = 2.

Three-dimensional T1 -weighted MR images were acquired by a gradient-recalled echo sequence with the following acquisition parameters: TR/TE = 12 ms/6 ms, scan time = 3.09 min, FOV = 7 cm × 7 cm, matrix = 256 × 256, ST = 1 mm, spatial resolution = 0.27 mm × 0.27 mm × 1 mm, interslice distance = 1 mm, number of slices = 60, and NA = 1, flip angle = 15°.

For the DTI acquisition, diffusion weighted images were acquired with TR/TE = 4,000 ms/86 ms, FOV = 6 cm × 6 cm, ST = 1.8 mm, inter-slice distance = 2 mm, matrix = 64 × 64, in-plane voxel size = 234 μm × 234 μm, NA = 1, *b*-value = 0 and 1,000 s/mm^2^ applied in 15 non-colinear directions.

### Immunostaining

Harvested brain samples of rats were post-fixed in 4% PFA for 24 h, and then were vitrified in 20 and 30% sucrose solutions for 24 h and 3 days, respectively. Coronal brain sections from all subjects were obtained using a cryostat (RM2135, Leica, Wetzlar, DE). IF was performed on cryosections (20 μm): approximately 1.70 to -4.80 mm to Bregma according to Paxinos and Watson (1998). In details, brain sections were washed three times with PBS (pH = 7.4). Sections were then blocked from non-specific binding with 10% normal donkey serum in PBS containing 0.3% Trition-X-100 (Sigma-Aldrich, St. Louis, MO, United States) for 2 h at room temperature. Primary antibodies used were: (1) rabbit polyclonal anti-NICD (Abcam, Cambridge, MA, United States; 1:100); (2) mouse monoclonal anti-Nestin (BD Biosciences, Franklin Lakes, NJ, United States; 1:1000); (3) goat monoclonal anti-Dcx (Santa Cruz Biotechnology, Santa Cruz, CA, United States; 1:100); (4) rabbit monoclonal anti-NeuN (Biosensis, Thebarton, SA, Australia; 1:250); (5) mouse polyclonal-anti MBP (Abcam, Cambridge, MA, United States; 1:500). Primary antibodies were incubated overnight with sections at 4°C and balanced at room temperature for about 30 min. Sections were then rinsed with PBS 3 times for 5 min and incubated with the following secondary antibodies: Alexa Fluor 488- and 568- conjugated donkey anti-mouse, anti-rabbit, or anti-goat (Life Technologies, Carlsbad, CA, United States; 1:200). Sections were subsequently rinsed with PBS 3 times for 5 min and counterstained with DAPI, nuclear stain (Sigma-Aldrich, St. Louis, MO, United States; 1:1000). Finally, all the sections were rinsed with PBS 3 times for 5 min, and coverslipped with mounting medium. Sections from the different groups were respectively processed together in the same batches to minimize staining variability.

### Image Processing and Quantitative Analysis

#### Magnetic Resonance Imaging

Regions of interest (ROIs) including the STR and the IC were selected for analysis of T1 SI or DTI parameters. For T1 SI analysis of STR, the ROI was placed on the striatal ischemic boundary with the T1 MR image, which includes the largest area of the lesion.

To assess the DTI parameters, axial T2 MR images were used for anatomical references to assess the topography of infarction. DTI measurements including FA, axial diffusivity (AD) and radial diffusivity (RD) were analyzed in the ischemic boundary of STR, as well as the ipsilateral IC. Contralateral ROI regions were drawn on the contralateral hemisphere according to the size and shape of the ipsilateral ROIs. The entire ROI analysis was repeated twice to ensure reproducibility of the results. The percent change of every parameter value was computed as: 100 × (X_i_ - X_c_) / X_c_, where X represents an averaged metric.

#### Immunostaining

For histological images, sections were scanned with a × 20 primary objective of a Vslide scanning microscope (Nikon, Chiyoda, Tokyo, Japan) with filter sets for DAPI (EX350/50- EM470/40), FITC (EX493/16-EM527/30), and FRITC (550,620). The initial captures were stitched into the Vslide software to create the integrated images of the whole brain. All the images were acquired with a resolution of 1024 × 1024 pixels using constant values for laser power, pinhole, digital gain, offset, and scan speed. With Image J (National Institutes of Health, Bethesda, MD, United States), the acquired images were semi-quantitatively measured by optical density of positively stained cells of the selected ROI. In sham-operated and ischemic rats, firstly, the intensity of NICD, Nestin, Dcx of SVZ and STR from 1 day to 7 days was calculated. In vehicle- and DAPT-treated animals, the intensity of Dcx was calculated in the ipsilateral STR using a 0.5-mm-wide quadratic grid. The intensity of NeuN and MBP in the ischemic boundary of STR and the intensity of MBP in the ipsilateral IC were calculated. Each value was also calculated in the contralateral site equivalent to the ipsilateral ROIs and was also computed as: 100 × (X_i_ - X_c_) / X_c_, where X represents an averaged metric.

#### Functional Examination

Vehicle- and DAPT-treated animals were subjected to behavioral tests to assess sensorimotor function 2, 3, 4, and 8 weeks after the induced stroke. The neurological examination had a maximum of 42 points and 0 in normal rats, including postural signs (forelimb flexion, thorax twisting), gait disturbances (circling, climbing, biased movement when pulling the tail or pushing the back), limb placing (forelimb, hindlimb), beam balance, symmetry of muscle tone and strength, sensory function, and spontaneous activity (Reglodi et al., 2003).

### Statistical Analysis

One-way analysis of variance was performed for multiple group comparisons with *post hoc* LSD tests performed for each of the two groups. Unpaired *t*-tests were performed for two groups comparison at different time points. The correlations between functional test and histological changes were assessed using the Pearson Product correlation analysis. In statistical tests, differences were considered significant when *P* < 0.05 and data were presented as the mean ± SD. The statistical analysis was performed using Prism, version 6.0 (GraphPad Software Inc., La Jolla, CA, United States).

## Results

### Notch1 Signal, NSCs, and Neuroblasts Responses After Stroke

#### Activation of Notch1 Signaling

**FIGURE 2:**
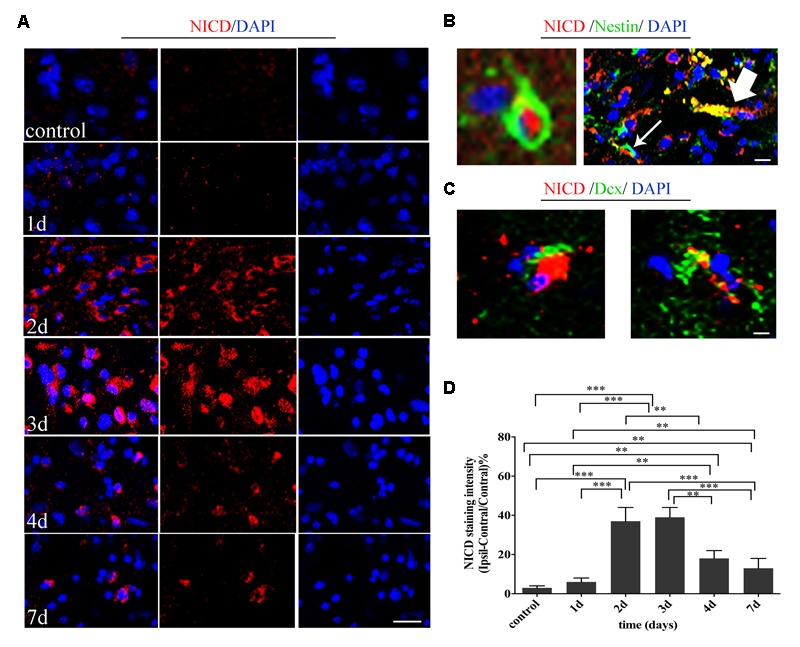
Temporal evolution and spatial location of Noth1 signaling in ischemic striatum (STR). **(A)** Immunostaining of NICD before and at day 1, day 2, day 3, day 4, and day 7 after MCAO. Scale bar = 20 μm. **(B)** Double immunostaining of NICD/Nestin at the ischemic boundary and the lesion of day 3 after MCAO. The thin white arrow and thick white arrow show the double location of the branched neural stem cell (NSC) and vessel-like NSC respectively. Scale bar = 10 μm. **(C)** Double immunostaining of NICD/Dcx at the ischemic boundary of day 4 after MCAO. Scale bar = 10 μm. **(D)** Quantification of NICD fluorescence intensity in ischemic STR. ^∗∗∗^*P* < 0.001 versus day 2, day 3 and control, day 1, day 7. ^∗∗^*P* < 0.01 versus day 4 and control, day 1, day 2, day 3. ^∗∗^*P* < 0.01 versus day 7 and control, day 1.

First, we evaluated the activity of Notch1 signaling at the acute and subacute phase of ischemia by histochemical analysis of the NICD from day 1 to day 7 after the induced stroke (**Figure 2A**). The intensity of NICD was significantly elevated 2 and 3 days after reperfusion (versus sham-operated group and 1 day, *P* < 0.001), and reversely decreased 4 days (versus 2 and 3 days, *P* < 0.01) and 7 days later (versus 2 and 3 days, *P* < 0.001) later (**Figure 2D**). Next, to assess whether Notch1 signaling was activated in neural progenitors and neuroblasts after MCAO, we double stained the harvested tissue for markers of Notch intracellular domain (NICD), NSCs (Nestin) and neuroblasts (Dcx). Our results showed insignificant staining for NICD, Nestin and Dcx in sham-operated rats, while in ischemic rats, NICD (+) cells were increased in the ipsilateral STR, where a few of NICD/Nestin (+) cells and NICD/Dcx (+) cells were detected 3 and 4 days after reperfusion. In addition, the NICD was found to be expressed in both the body and the branches of the branched Nestin (+) cells (**Figure 2B**, thin white arrow) or Dcx (+) cells (**Figure 2C**), as well as in the vessel-like Nestin (+) cells (**Figure 2B**, thick white arrow), as indicated in the representative images taken 4 days after reperfusion.

### Distribution and Expression of Nestin and Dcx

**FIGURE 3:**
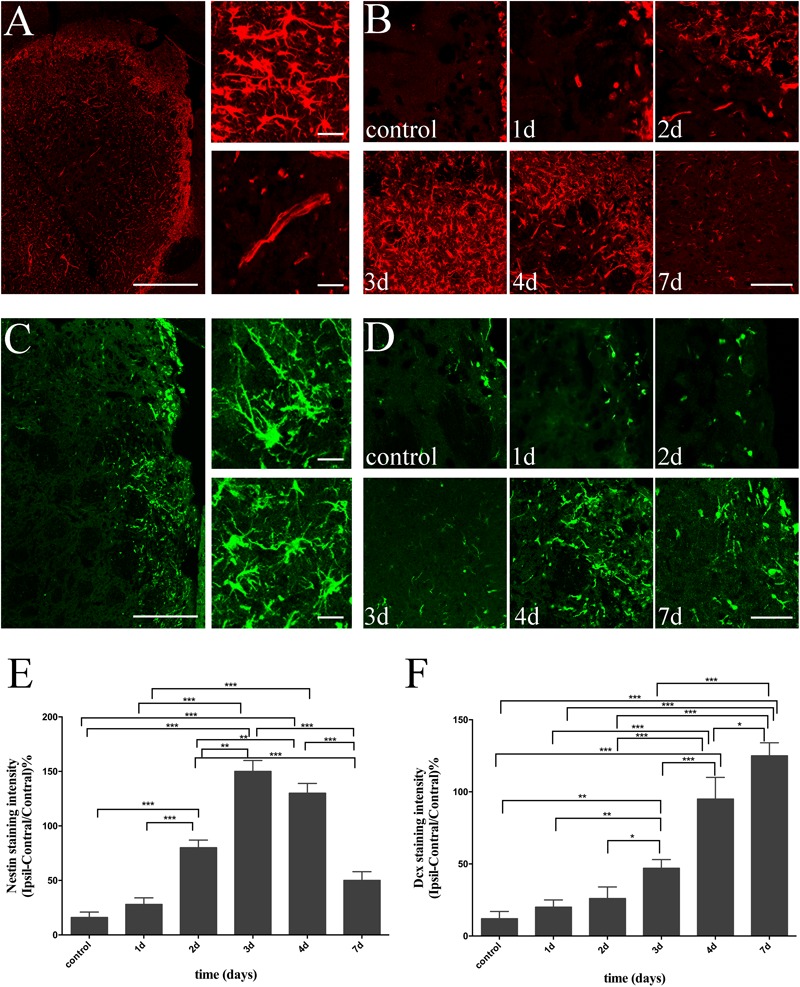
Temporal evolution of NSCs and immature neurons in ischemic STR. **(A)** The “photomerged” image shows the location of Nestin (+) cells in ischemic STR. The corresponding right panel presents × 20 magnification images of branched and vessel-like NSCs respectively. Scale bar = 100 and 20 μm. **(B)** Immunostaining of Nestin before and at day 1, day 2, day 3, day 4 and day 7 after MCAO. Scale bar = 50 μm. **(C)** The “photomerged” image shows the location of Dcx (+) cells in ischemic STR. The corresponding right panel presents × 20 magnification images of immature neurons be characteristic of elongated processes or riches in processes in different directions respectively. Scale bar = 100 and 20 μm. **(D)** Immunostaining of Dcx before and at day 1, day 2, day 3, day 4, and day 7 after MCAO. Scale bar = 50 μm. **(E)** Quantification of Nestin fluorescence intensity in ischemic STR. ^∗∗∗^*P* < 0.001 versus day 2, day 3, day 4 and control, day 1, day 7. ^∗∗^*P* < 0.001 versus day 2 and day 3, day 4. **(F)** Quantification of Dcx fluorescence intensity in ischemic STR. ^∗∗∗^*P* < 0.001 versus day 4, day 7 and control, day 1, day 2, day 3. ^∗∗^*P* < 0.01 versus day 3 and control, day 1. ^∗^*P* < 0.05 versus day 3 and day 2. ^∗^*P* < 0.05 versus day 7 and day 4.

At the same time, we investigated the spatial and temporal evolution of NSCs and neuroblasts following ischemia by analyzing the expression of Nestin and Dcx from day 1 to day 7 after MCAO. Nestin, a specific marker of NSCs, was almost non-detectable in the non-ischemic brain, but present in the lesion core (vessel-like structure), as well as in the ischemic boundary (branched structure) in ischemic brain slices (**Figure 3A**). Moreover, the intensity of Nestin was statistically elevated 2 days (versus Control, 1 day, *P* < 0.001), peaked at 3 days (versus control, 1 day, *P* < 0.001; versus 2 days, *P* < 0.01), and statistically decreased 7 days (versus 3 and 4 days, *P* < 0.001) following reperfusion (**Figures 3B,E**). Dcx, a specific marker for neuroblasts and differentiating neurons, was detected in the SVZ and in a few striatal cells in sham-operated rats. There were both migrating and non-migrating Dcx (+) cells induced by cerebral ischemia, with the former one being characteristic of elongated and leading processes, while the later one was rich in processes growing in different directions (**Figure 3C**). Surprisingly, as time followed, we found that migrating Dcx (+) cells were highly distributed in the ischemic STR with their leading processes directed away from the SVZ (**Figure 3D**). The intensity of Dcx was statistically elevated 3 days later (versus control, 1 day, *P* < 0.01; versus 2 days, *P* < 0.05), significantly increased 4 days later (versus control, 1, 2, and 3 days, *P* < 0.001), and persisted for 7 days (versus control, 1, 2, and 3 days, *P* < 0.001; versus 4 days, *P* < 0.05) after stroke in the observation time (**Figures 3D,F**).

### Reorganization of White Matter After DAPT Treatment

#### T1 SI Changes in Ischemic Boundary

**FIGURE 4:**
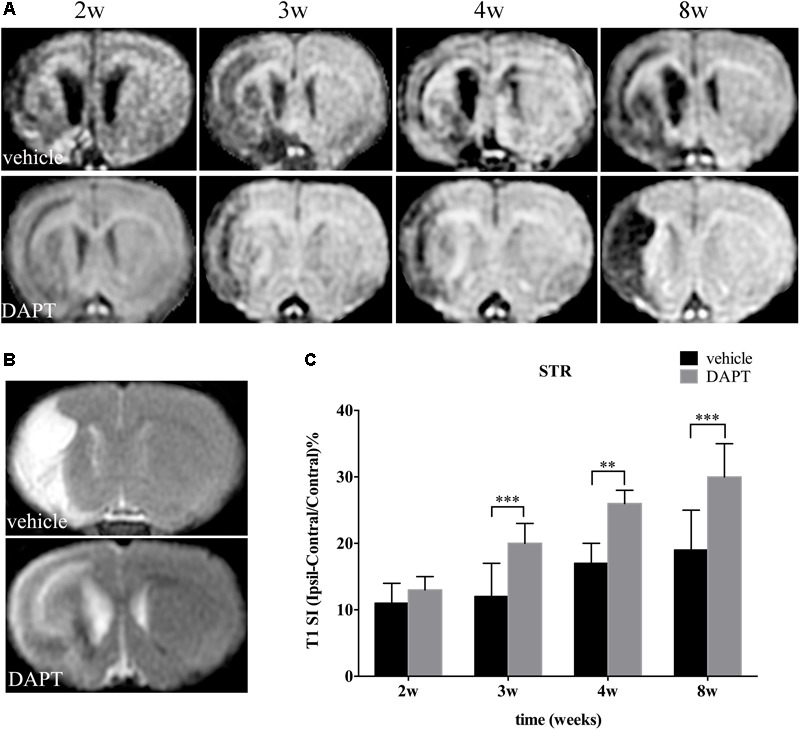
Effects of DAPT on structure changes of STR with T1 MRI after MCAO. **(A)** The evolution changes in T1 MR images in DAPT-treated rats and vehicle rats from 2 to 8 w. **(B)** T2 MR images of DAPT-treated rats and vehicle rats at 8 w. **(C)** Comparison of T1 signal intensity of ischemic boundary between two group rats. ^∗∗∗^*P* < 0.001, 3 w; ^∗∗^*P* < 0.01, 4 w; ^∗∗∗^*P* < 0.001, 8w versus DAPT and vehicle.

Measurements of T1 SI were performed on the ischemic boundary of STR, delineated according to the ischemic lesions in T2 MR images, in ischemic animals treated with or without DAPT (**Figures 4A,B**). Notably, there was no difference of T1 SI between DAPT-treated versus vehicle-treated animals 2 weeks after the induced stroke, and their differences became significant 3 weeks (*P* < 0.001), 4 weeks (*P* < 0.01), and 8 weeks (*P* < 0.001) following cerebral ischemia (**Figure 4C**).

#### DTI Parameter Changes of CST

**FIGURE 5:**
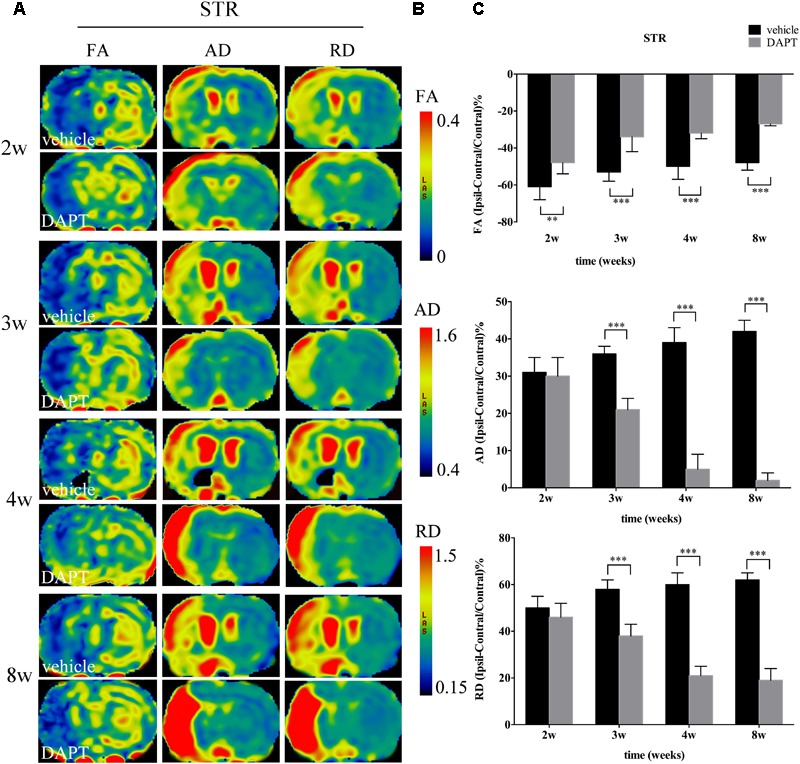
Effects of DAPT on microstructure changes of ischemic boundary of STR with DTI maps after MCAO. **(A)** The evolution changes in FA, AD and RD of ipsilateral STR in DAPT-treated rats and vehicle rats from 2 to 8w. **(B)** The range of FA, AD, and RD. **(C)** Comparisons of FA, AD, and RD of ischemic boundary of STR between two group rats. FA: ^∗∗^*P* < 0.01, 2w; ^∗∗∗^*P* < 0.001, 3w, 4w, 8w versus DAPT and vehicle. AD, RD: ^∗∗∗^*P* < 0.001, 3w, 4w, 8w versus DAPT and vehicle.

**FIGURE 6:**
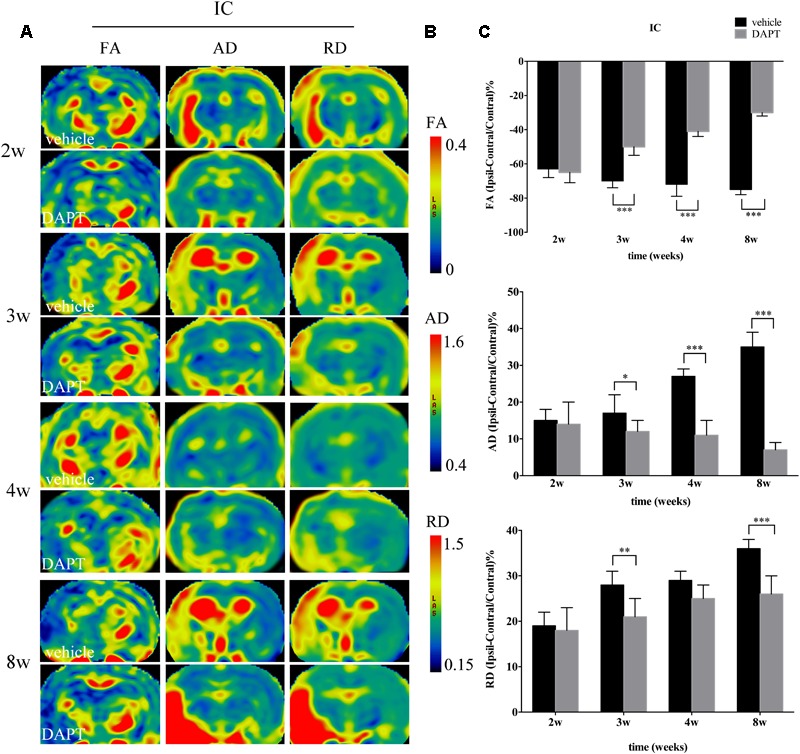
Effects of DAPT on microstructure changes of internal capsule (IC) with DTI maps after MCAO. **(A)** The evolution changes in FA, AD and RD of ipsilateral IC in DAPT-treated rats and vehicle rats from 2 to 8w. **(B)** The range of FA, AD and RD. **(C)** Comparisons of FA, AD and RD of ipsilateral IC between two group rats. FA: ^∗∗∗^*P* < 0.001, 3w, 4w, 8w versus DAPT and vehicle. AD: ^∗^*P* < 0.05, 3w; ^∗∗∗^*P* < 0.001, 4w, 8w versus DAPT and vehicle. RD: ^∗∗^*P* < 0.01, 3w; ^∗∗∗^*P* < 0.001, 8w versus DAPT and vehicle.

Diffusion tension imaging measurements of FA, AD, and RD were performed on the ischemic boundary of STR and ipsilateral IC (**Figures 5A, 6A**), with a consistent range for each value in the two groups (**Figures 5B, 6B**). In the ischemic boundary zone of STR, FA maps showed a gradual increase in FA at different time points of DAPT- and vehicle-treated rats with differences being significant at 2 weeks (*P* < 0.01). This increase significantly expanded with longer follow-up time points (i.e., from 3 to 8 weeks, *P* < 0.001) following reperfusion (**Figure 5C**). AD and RD measurements exhibited similar temporal profiles, which differed from FA, that AD and RD measurements in the ipsilateral ROIs were higher than those in the contralateral side during our observation time in both DAPT- and vehicle-treated animals. However, AD and RD represented a gradual increase in vehicle-treated subjects, but a decrease in DAPT-treated group during our observation time period. Importantly, statistically significant differences were detected between two the groups (both AD and RD, from 3 to 8 weeks, *P* < 0.001) (**Figure 5C**).

In the ipsilateral IC, FA maps showed opposite temporal profiles between the two subject groups with FA gradually decreasing in the vehicle-treated group while increasing in the DAPT-treated group, showing significant differences from 3 to 8 weeks (*P* < 0.001) (**Figure 6C**). Similar to what was observed in the STR, the AD and RD measurements in the ipsilateral IC were higher than those of the contralateral hemisphere in both groups during our observation time. AD was gradually increased in the vehicle-treated group but decreased in the DAPT-treated one with these differences becoming significant at 3 weeks (*P* < 0.05), expanding at 4 weeks (*P* < 0.001), and 8 weeks (*P* < 0.001) following reperfusion (**Figure 6C**). At the same time, RD gradually increased in the two groups, but changed slowly in the DAPT-treated group with statistically significant differences at 3 weeks (*P* < 0.01) and 8 weeks (*P* < 0.001) following reperfusion (**Figure 6C**).

### Stroke-Generated Neurons in Ischemic Boundary

**FIGURE 7:**
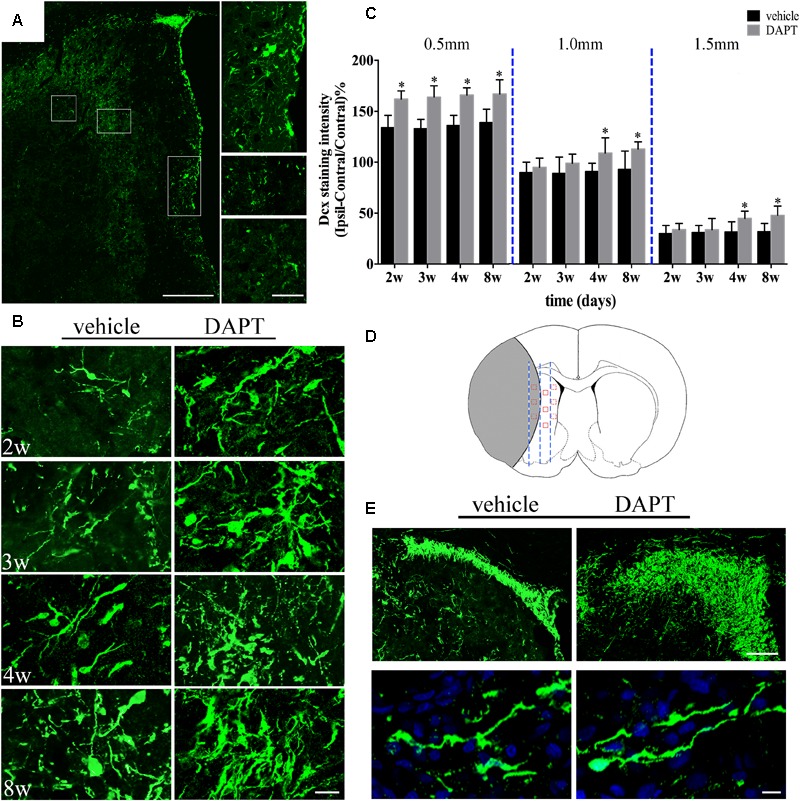
Effects of DAPT on neuroblasts activity after MCAO. **(A)** Boxes in the “photomerged” image shows the distribution of Dcx (+) cells across the ischemic boundary to the lesion. Scale bar = 100 and 50 μm. **(B)** Immunostaining of Dcx inside 0.5mm distances from the SVZ in two group rats from 2 to 8w. Scale bar = 20 μm. **(C)** Comparisons of Dcx fluorescence intensity in two group rats at different distances from the SVZ 2, 3, 4, and 8 weeks after MCAO. 0.5 mm: ^∗^*P* < 0.05, 2, 3, 4, 8w versus DAPT and vehicle. 1.0 mm, 1.5mm: ^∗^*P* < 0.05, 4w, 8w versus DAPT and vehicle. **(D)** Anatomical references showing the ROIs. **(E)** Representative images of the location of Dcx (+) cells in the anterior SVZ and shape of Dcx (+) cells in the ischemic boundary of two group rats 8 weeks after MCAO. Scale bar = 50 and 10 μm.

To confirm whether the improved structural changes in the MR images were attributed to the favored neurogenesis by suppression of Notch1 signaling, we used the marker of Dcx and NeuN (a marker of mature neurons) on the ischemic boundary in both vehicle- and DAPT-treated groups at the early and late periods of the chronic stage of stroke respectively. Dcx (+) cells were found distributed from the SVZ laterally toward the damaged region and presented long lasting processes in all subjects, as seen in the corresponding representative image (**Figure 7A**). Moreover, the SVZ area in the DAPT-treated group presented more Dcx (+) cells, 2–8 weeks after stroke induction (**Figure 7B**). To detect the effect of DAPT treatment on the proliferation and migration of neuroblasts, we calculated the intensity of Dcx at 0.5, 1.0, and 1.5 mm from the SVZ (**Figures 7C,D**). Our results showed that DAPT treatment increased Dcx (+) cells within 0.5 mm from 2 to 8 weeks (*P* < 0.05), 1.0 mm at 4 weeks, 8 weeks (*P* < 0.05), and 1.5 mm at 4 weeks, 8 weeks (*P* < 0.05). Furthermore, 8 weeks after reperfusion, the DAPT-treated animals harbored more Dcx (+) cells in the anterior SVZ (aSVZ) and presented identical structure of Dcx (+) cells in the ipsilateral STR (**Figure 7E**).

**FIGURE 8:**
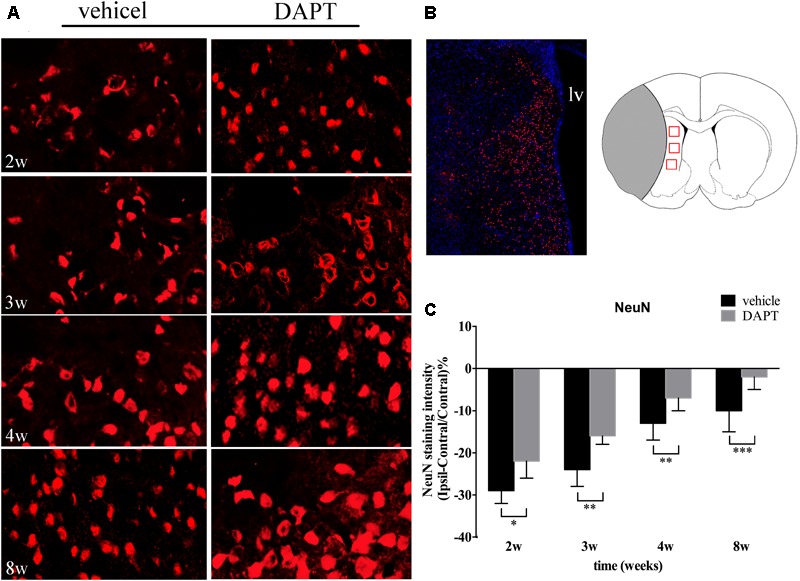
Effects of DAPT on mature neurons activity after MCAO. **(A)** Immunostaining of NeuN in ischemic boundary of STR in two group rats from 2 to 8w. Scale bar = 20 μm. **(B)** The “photomerged” image shows the location of NeuN (+) cells in the ischemic boundary of STR. The anatomical reference shows the ROIs. Scale bar = 100 μm. **(C)** Comparisons of NeuN fluorescence intensity in two group rats. ^∗^*P* < 0.05, 2w; ^∗∗^*P* < 0.01, 3w, 4w; ^∗∗∗^*P* < 0.001, 8w versus DAPT and vehicle.

We next analyzed the intensity of NeuN staining in the ischemic boundary in both groups (**Figures 8A,B**). Our results showed that the number of neurons in the DAPT-treated animals increased when compared to the vehicle-treated group, 2 weeks (*P* < 0.05), 3 weeks (*P* < 0.01), 4 weeks (*P* < 0.01), and 8 weeks (*P* < 0.001) after stroke (**Figure 8C**).

### Remyelination of CST

**FIGURE 9:**
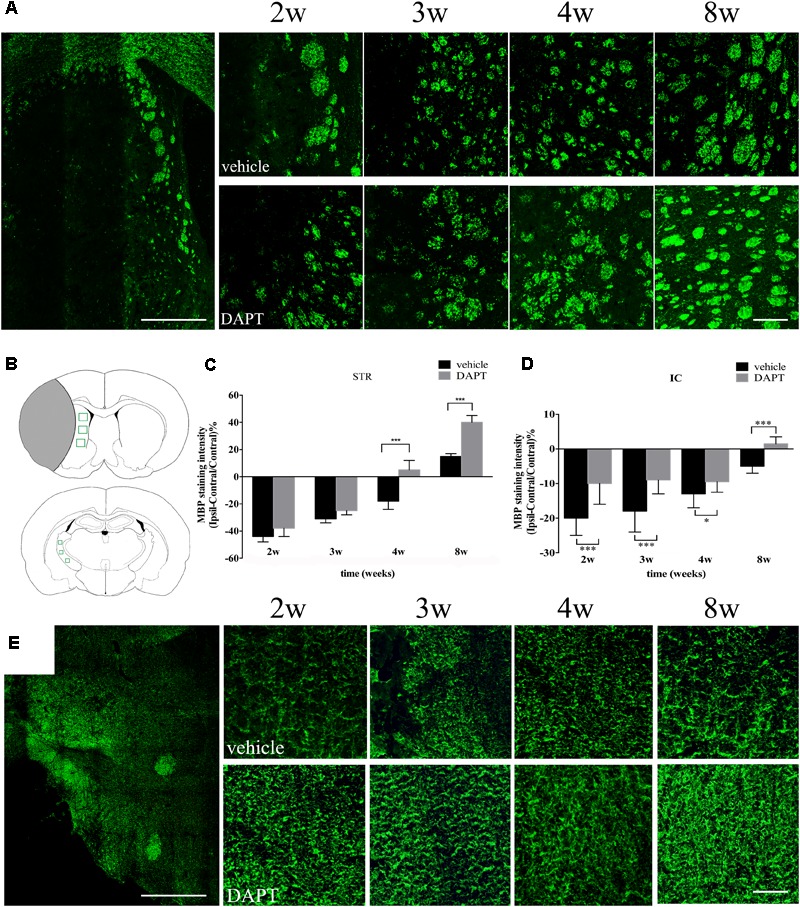
Effects of DAPT on MBP immunoreactivity of STR and IC after MCAO. **(A)** The “photoemrged” image shows the location of MBP (+) cells in the ipsilateral STR. Immunostaining of MBP in ischemic boundary of STR in two group rats from 2 to 8w. Scale bar = 100 and 20 μm. **(B)** Anatomical references show ROIs in ipsilateral STR and IC respectively. **(C)** Comparisons of MBP fluorescence intensity of ischemic boundary of STR in two group rats. ^∗∗∗^*P* < 0.001, 4w, 8w versus DAPT and vehicle. **(D)** Comparisons of MBP fluorescence intensity of ipsilateral IC in two group rats. ^∗∗∗^*P* < 0.001, 2, 3, 8w; ^∗^*P* < 0.05, 4w versus DAPT and vehicle. **(E)** The “photoemrged” image shows the location of MBP (+) cells in the ipsilateral IC. Immunostaining of MBP in ipsilateral IC in two group rats from 2 to 8w. Scale bar = 100 and 20 μm.

Since newborn neurons play protective roles on the growing axons, we further analyzed the immunoreactivity of the MBP (a protein present in the myelin sheath surrounding axons) in neural pathways of the STR and the IC. In the ipsilateral STR, bundles of axons were mainly located at the ischemic boundary (**Figure 9A**). The intensity of MBP gradually increased with time in both groups (**Figure 9A**), which was larger in the DAPT-treated group, showing statistically significant differences at 4 weeks (*P* < 0.001) and 8 weeks (*P* < 0.001) after reperfusion (**Figures 9B,C**). In the ipsilateral IC, axons were presented as fibrous structures, which were positive for MBP antibody (**Figures 9B,E**). And the intensity of the MBP signal increased following DAPT treatment at 2 weeks (*P* < 0.001), 3 weeks (*P* < 0.001), 4 weeks (*P* < 0.05), and 8 weeks (*P* < 0.001) after reperfusion (**Figures 9D,E**).

### Correspondence Between MRI and Histological Measures

**FIGURE 10:**
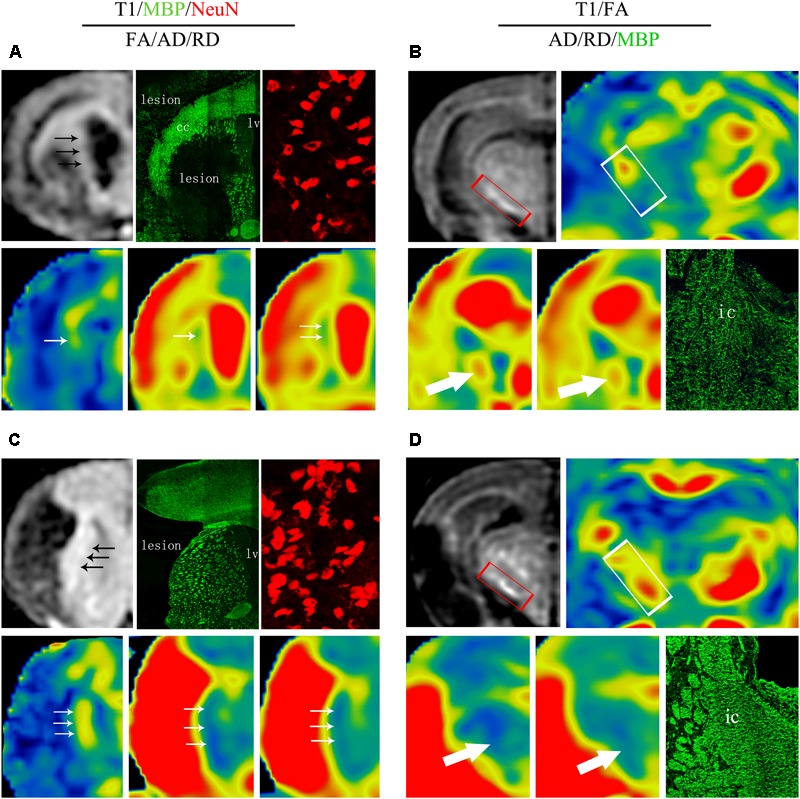
Comprehensive analysis about effects of DAPT on structure-based and cell-based changes in ipsilateral STR and IC 8 weeks after MCAO. **(A)** Representative images of T1 MR, MBP, NeuN, FA, AD and RD of ischemic boundary in vehicle rats. **(B)** Representative images of T1 MR, FA, AD, RD, and MBP of ipsilateral IC in vehicle rats. **(C)** Representative images of T1 MR, MBP, NeuN, FA, AD, and RD of ischemic boundary in DAPT-treated rats. **(D)** Representative images of T1 MR, FA, AD, RD and MBP of ipsiateral IC in DAPT-treated rats.

The relationships between the reorganized tissue across the ipsilateral CST detected by MRI and its corresponding histological changes at 8 weeks are presented in **Figure 10**. In the DAPT treated group, the ischemic boundary of the STR presented a higher T1 SI, as well as an early and larger recovery of FA, AD, and RD measurements, in accordance with an increased number of mature neurons and axon bundles (**Figures 10A,C**). At the same time, the ipsilateral IC presented an early and larger recovery of FA and AD, as well as a moderate increase of RD, in accordance with an increase of axonal bundles in the DAPT-treated group (**Figures 10B,D**).

### Functional Status

**FIGURE 11:**
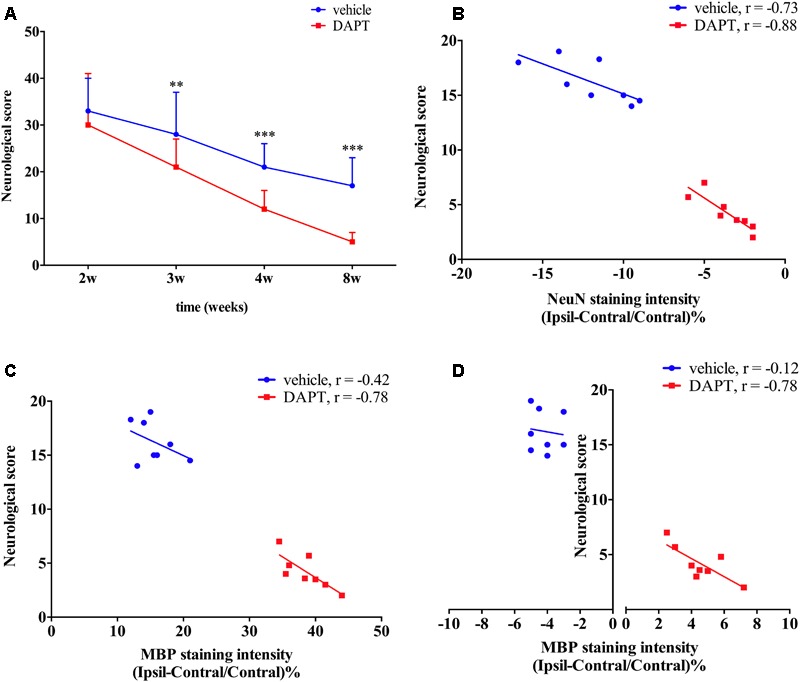
Functional test and correlation analysis. **(A)** Neurological score. **(B)** The correlation analyze between neurological score and NeuN staining intensity. **(C)** The correlation analyze between neurological score and MBP staining intensity of ipsilateral STR. **(D)** The correlation analyze between neurological score and MBP staining intensity of ipsilateral IC.

At the same time, we explored whether functional recovery after stroke occurs concomitantly with the increased number of mature neurons in the ischemic boundary of the STR and the reorganization of myelin sheaths in the ipsilateral CST following DAPT treatment. Functional test examinations showed that animals subjected to DAPT-treatment presented an early and larger motor functional improvement with time (3 weeks, *P* < 0.01; 4 and 8 weeks, *P* < 0.001) (**Figure 11A**). We further analyzed the correlation between functional outcomes at 8 weeks and the corresponding changes in the number of mature neurons as well as the myelin reorganization presented. Our analysis showed a negative correlation between the neurological score and the NeuN staining intensity in the STR in both groups examined, with the differences being more significant in the DAPT-treated group (vehicle, *r* = -0.73, *P* < 0.05; DAPT, *r* = -0.88, *P* < 0.01) (**Figure 11B**). At the same time, negative correlations were found between neurological scores and MBP staining intensity in the STR (*r* = -0.78, *P* < 0.05) and the IC (*r* = -0.78, *P* < 0.05) in the DAPT-treated group but not in vehicle-control (**Figures 11C,D**).

## Discussion

In this study, we first investigated the temporal evolution of Notch1 signaling at the early stage of stroke. Notch1 signaling was found to be maximally activated 3 days after ischemia induction and was reversely attenuated 4 and 7 days after reperfusion. This result is in accordance with the previous study indicating that Notch1 signaling was activated in the acute phase of stroke and then gradually declined in the subacute stage (Oya et al., 2009). Next, we detected the location of NICD in Nestin- and Dcx-positive cells by immunohistochemical analysis, indicating that Notch1 signaling was activated in this cell population and may regulate endogenous neurogenesis.

At the same time, we detected the spatial and temporal evolution of NSCs and neuroblasts at the early stage of stroke. Nestin-positive cells were found to be diffusively located at the lesion and the peri-lesion area of the ipsilateral STR and the ipsilateral cortex. In the ipsilateral STR, there was little expression of Nestin at day 1, which was statistically increased at day 2, peaked at day 3, while reversely declined at day 4 and day 7 following reperfusion. Dcx-positive cells were located at the ipsilateral STR, but not at the ipsilateral cortex, which was consistent with previous studies (Arvidsson et al., 2002). The expression of Dcx statistically significantly increased at day 3 and increased at day 4, day 7 after stroke, which exhibited chains oriented toward the ischemic boundary, formed clusters, and were dispersed in the ipsilateral striatum. Compelling evidences have previously shown that actively proliferating progenitor cells in the SVZ differentiate into migrating neuroblasts that travel to the ischemic boundary (Arvidsson et al., 2002; Thored et al., 2006). The above temporal expression of Nestin and Dcx at the early stage of stroke in our study showed the largest proliferative period of NSCs and the statistically significant increases in neuroblast proliferation at day 3, indicating the potential transdifferentiation from NSCs to a neuronal lineage. Additionally, the temporal migration of neuroblasts from the SVZ to the ischemic striatum also corresponds to a tendency for neuronal regeneration (Arvidsson et al., 2002; Thored et al., 2006).

Recent studies have demonstrated that early activation of Notch1 signaling activation promotes NSCs proliferation, while late Notch1 signaling activation promotes NSCs differentiation (Oya et al., 2009; Wang L. et al., 2009). The results in our study indicated that the temporal profile of NICD is comparable with the expansion of neural progenitors and the generation of neuroblasts after stroke (Oya et al., 2009; Wang L. et al., 2009). Importantly, blocking Notch1 signaling activation at the appropriate time to expand neural progenitor pools and augment neural differentiation may be a therapeutic intervention with significant benefits to stroke patients. Thus, based on the results of our current study, we hypothesized that a proper therapeutic time point may be 4 days following stroke in this rat model.

In our previous study, we demonstrated that Notch1 signaling could be blocked by DAPT at day 4 after stroke (Hao et al., 2017). Based on this work, we used the same method in this study, to evaluate microstructural changes of rat brains *in vivo* at chronic stages of stroke by monitoring a set of MRI parameters in the CST both proximal and distal to the ischemic lesion. DTI measurements alone are not sufficient to tackle the complex pathology underlying ischemic processes, additional contrasts, such as T1 and T2 MRI images, should be included in future study designs, together with an integration of the data presented here, could provide complementary information about the status of ischemic tissue following stroke (Jacobs et al., 2001; Wegener et al., 2006). In this study, we firstly analyzed the T1 MR images to distinguish and stage the ischemic boundary changes, and further characterized the microstructure details with both the T1 SI and diffusion MR parameters (FA, AD, RD). The ischemic boundary in the STR presented a gradual increase of T1 SI in all ischemic rats during the chronic stage after stroke, which accounted for various important factors including decreased edema, astrogliosis, and microglial infiltration, as well as neurogenesis and remyelination. Particularly, the larger increase of T1 caused by the DAPT treatment may account for one of the factors mentioned above (Wegener et al., 2006). To further detect the microstructure changes of white matter bundles, we analyzed the diffusion changes of CST, which may allow the prediction of functional outcomes in chronic stroke patients (Stinear et al., 2007). FA is a common DTI parameter that measures the degree of voxel diffusion (Jones et al., 2013; Ferris et al., 2017). AD quantifies diffusivity along the major orientation of the axon bundles (Song et al., 2002), while RD represents diffusivity perpendicular to white matter bundles (Suttner et al., 2016). When a tissue undergoes ischemia, structural networks represent two signatures including perilesional degenerative changes and remote transneuronal changes, which are both characterized by decreased FA and increased AD, RD measurements (Umarova et al., 2017). These changes are accompanied with histological changes including neural shrinkage as well as a reduction of axonal myelin content and fiber number (Umarova et al., 2017). In our study, FA gradually increased in the ipsilateral STR, while decreased in the ipsilateral IC following ischemia, showing a larger increase in the STR and an early increase in the IC after DAPT treatment. AD was increased in both the ipsilateral STR and the IC after stroke, and gradually decreased to baseline levels following DAPT treatment. Similarly, RD increased in the ipsilateral STR and the IC after ischemia, and showed early decrease in the STR, as well as a moderate increase in the IC after DAPT treatment. The relative recovery of these diffusion parameters mirrored previously reported motor functional abilities of ischemic rats after DAPT treatment (Puig et al., 2010; Reijmer et al., 2013; Feng et al., 2015; Bigourdan et al., 2016), which are now corroborated with our study, which showed that motor function improved after DAPT treatment when compared to vehicle controls in a rat model of stroke.

Using histological examination, we found that neuroblasts were distributed laterally from the SVZ to the ischemic lesion. At different distances from the SVZ, the intensity of Dcx was increased within 0.5 mm from 2 to 8 weeks, and increased within 1.0 and 1.5 mm at 4 and 8 weeks, indicating a promotion in neuroblast proliferation during the entirety of our observation period, as well as the induction of cell migration during the late period. DAPT-treated animals presented a large potential for neurogenesis, harboring an increase number of neuroblasts in the aSVZ. More importantly, our two treated groups presented identical structures of neuroblasts, which suggests that DAPT treatments may not alter the function of neuroblasts. Furthermore, the NeuN staining examination showed an increase of mature neurons in the ischemic boundary of the STR in the DAPT-treated group. Significantly, the MBP staining examination showed that the integrity of the CST was recovered early in both the ischemic boundary and the ipsilateral IC.

In this study, though there was no attenuation of necrosis across the lesion, the motor outcome in the DAPT-treated group was significantly elevated when compared to the vehicle-treated group. The most possible contribution should be attributed to the promotion of neurogenesis following DAPT treatment. First, these newborn striatal neurons integrate with preexisting neurons to form functional synapses in the viable brain tissue (Hou et al., 2008; Sun et al., 2012). Secondly, these newly generated neurons could form functional long axons projecting to remote regions to participate in the restorative processes of brain tissue (Sun et al., 2012). As target regions of CST, the STR and the IC play critical roles in the regulation of motor behavior, in which an increase in neural connections could replace damaged ones and ameliorate motor outcomes after stroke. From a novel and comprehensive perspective, with the non-invasive method of MRI, we provided morphological and functional evidence that DAPT treatment could ameliorate the integrity of axon bundles. The post-mortem histological examination confirmed the increase in numbers of mature neurons in the ischemic boundary zone and the promotion of remyelination across the CST.

With DAPT treatment, the increase in numbers of mature neurons and the ultimately improved motor outcome may be attributed to the attenuated inflammatory responses (Hao et al., 2017), which had been demonstrated in our previous work, and the promoted neurogenesis, which was suggested in the current study. Adult neurogenesis following stroke is a sequential and multistep process ranging from neural precursor proliferation, migration toward the ischemic site, and differentiation into the proper neural phenotype (Bonfanti and Peretto, 2007; Oya et al., 2009; Christie and Turnley, 2012). In this process, the Notch1 signaling pathway mediates expansion of the neural progenitor pool and neural differentiation, which is comparable to the temporal profile of NSCs (Wang L. et al., 2009). Inappropriate Notch1 activation may induce not only precocious maturation, but also a developmental stagnation of progenitors (Wang X. et al., 2009). Thus, the appropriate timely control of Notch1 signaling activity could produce a large proliferative pool and further promote neuronal differentiation.

## Conclusion

With this study, we provided additional evidence that the adult brain can undergo neural replacement from endogenous precursors to repair itself after stroke, which was shown here to be controlled by the activity of Notch1 signaling. Then with the non-invasive MR method, we detected the protective effects of DAPT treatment at the subacute stage of stroke to maximally promote endogenous neurogenesis and axonal reorganization. Our results may enhance the notion of Notch-1 based therapy efficacy in neural repair and promote its application in clinical trials.

## Author Contributions

X-ZH carried out the animal experiments, performed the MR scanning, and drafted the manuscript. L-KY processed the MR imaging and carried out the histological analysis. J-QT and C-CL processed the MR imaging. X-YF and Z-WY supervised the MR imaging. MJ performed the histological analysis. Y-MY instructed the study protocol and revised the manuscript. All of the authors read and approved the final manuscript.

## Conflict of Interest Statement

The authors declare that the research was conducted in the absence of any commercial or financial relationships that could be construed as a potential conflict of interest.

## Abbreviations

AD: axonal diffusion
ANOVA: One-way analysis of variance
aSVZ: anterior subventricular zone
CCA: common carotid artery
CST: corticospinal tract
DAPT: *N*-[*N*- (3,5-difluorophenacetyl)-1-alanyl]-S-phenylglycine t-butylester
Dcx: doublecortin
DTI: diffusion tension imaging
ECA: external cerebral artery
FA: fractional anisotropy
FOV: Field of View
IC: internal capsule
ICA: internal cerebral artery
IF: immunofluorescence
LSD: least-significant difference
LV: lateral ventricle
MBP: myelin basic protein
MCA: middle cerebral artery
MCAO: middle cerebral artery occlusion
MRI: magnetic resonance imaging
NA: number of average
NICD: Notch intercellular domain
NSC: neural stem cell
PBS: phosphate buffer saline
PFA: paraformaldehyde
RD: radial diffusion
ROI: region of interest
SD: standard deviation
SI: signal intensity
ST: slice thickness
STR: striatum
SVZ: subventricular zone
TE: echo time
TR: repetition time
